# Naturally Occurring Vegetation Connectivity Facilitates Ant-Mediated Coffee Berry Borer Removal

**DOI:** 10.3390/insects14110869

**Published:** 2023-11-10

**Authors:** Sanya Cowal, Jonathan R. Morris, Estelí Jiménez-Soto, Stacy M. Philpott

**Affiliations:** 1 Department of Environmental Studies, University of California Santa Cruz, Santa Cruz, CA 95064, USA; sphilpot@ucsc.edu; 2 School for Environment and Sustainability, University of Michigan, Ann Arbor, MI 48109, USA; jonno@umich.edu; 3 Geography, Environmental Science and Policy, School of Geosciences, University of South Florida, Tampa, FL 33612, USA; jimenezsoto@usf.edu

**Keywords:** connectivity, pest management, *Azteca* sp., vegetation structure, habitat complexity, coffee berry borer, agroecology, ants

## Abstract

**Simple Summary:**

The coffee berry borer (CBB), the most damaging coffee pest, significantly reduces coffee quality and yields. *Azteca* ants are known biological control agents of the CBB. Our paper demonstrates that the naturally occurring vegetation connectivity between coffee plants and *Azteca* nest trees facilitates ant mobility, resource recruitment, and CBB removal services. Promoting habitat conservation and structural complexity in agroecosystems supports ant-mediated ecosystem services and natural pest regulation.

**Abstract:**

Vegetation connectivity is an essential aspect of the habitat complexity that impacts species interactions at local scales. However, agricultural intensification reduces connectivity in agroforestry systems, including coffee agroecosystems, which may hinder the movement of natural enemies and reduce the ecosystem services that they provide. Ants play an important role in regulating the coffee berry borer (CBB), which is the most damaging coffee pest. For arboreal ant communities, the connections between trees are important structures that facilitate ant mobility, resource recruitment, foraging success, and pest control ability. To better understand how connectivity impacts arboreal ants in coffee agroecosystems, we conducted an experiment to assess the impact of artificial (string) and naturally occurring vegetation (vines, leaves, branches) connectivity on *Azteca sericeasur* behavior on coffee plants. We compared ant activity, resource recruitment, and CBB removal rates across three connectivity treatments connecting coffee plants to *A. sericeasur* nest trees: vegetation connectivity, string, and control (not connected) treatments. We found higher rates of ant activity, resource recruitment, and CBB removal on plants with naturally occurring vegetation connections to *A. sericeasur* nest trees. Artificial connectivity (string) increased the rates of resource recruitment and CBB removal but to a lesser extent than vegetation connectivity. Moreover, vegetation connectivity buffered reductions in ant activity with distance from the ant nest tree. These results reinforce how habitat complexity in the form of vegetation connectivity impacts interspecific interactions at the local scale. Our results also suggest that leaving some degree of vegetation connectivity between coffee plants and shade trees can promote ant-mediated biological pest control in coffee systems.

## 1. Introduction

Agricultural intensification simplifies ecosystems through management practices such as increases in agrochemical use, decreases in habitat complexity, and decreases in crop and vegetation diversity [1,2,3]. Agricultural intensification alters functional biodiversity; in particular, reductions in habitat complexity impact the arthropod community composition [4,5], decrease arthropod diversity [6,7,8,9] and reduce pest control services [10,11,12,13]. Notably, biological pest control is likely the ecosystem service most affected by biodiversity loss at the local scale [14].

In coffee agroecosystems, management intensification alters habitat complexity by impacting vegetation connectivity and structure [15,16,17]. The management intensification gradient ranges in coffee systems from the least intensive traditional shaded “rustic system”, in which coffee grows under a diverse closed canopy of native forest, to the most intensive “sun monoculture”, which refers to rows of open unshaded coffee monoculture, that require high inputs of agrochemicals [18,19,20]. On the shaded end of the intensification gradient, shade coffee habitats are naturally vegetatively complex, with diverse and dense shade canopies and vines and weeds that form connections between the shade trees and the coffee plants. This vegetation connectivity is an important aspect of habitat complexity that impacts species interactions at the local scale [21]. However, while progressing along the management intensification gradient, reductions in habitat complexity, driven by decreases in shade trees, increases in herbicide use, and the clearing of vegetation (weeds, epiphytes, and branches) between coffee plants, reduce vegetation connectivity and alter species interactions within ecological communities and the ecosystem services that they provide.

Connectivity is one physical component of habitats that has a profound impact on arboreal insects [22,23] and ant community structure [24,25]. In the absence of connectivity, trees are insular habitats with crown isolation that inhibits the movement of some taxa [26]. Connectivity in the form of lianas [24,25,26] and nylon ropes [27] shape the local community structure of arboreal ants, with higher ant species richness often occurring in trees that are connected artificially or vegetatively as compared with trees without these physical connections [26,27], and higher ant species coexistence occurring in trees with higher levels of naturally occurring canopy connectivity [22]. These results also reflect the nature of ants as highly efficient foragers, known to use branches and lianas as “opportunist walkways’’ that provide the quickest foraging routes by allowing for faster traveling speeds through avoiding obstacles (and hazards) on the ground [25,26], even if these routes are not necessarily the shortest distance [28]. The variation in texture of natural walkways, characterized as “surface roughness”, further impacts both arboreal and ground ant running speeds and foraging efficiency [25,26,29,30]. Physical connections between trees are thus important structures that facilitate not only arboreal ant mobility but also their foraging success, resource recruitment efficiency, and ant-provided ecosystem services, including pest removal [21].

Ants play an important role in the control of the coffee berry borer (*Hypothenemus hampei*, CBB), the most damaging insect pest of coffee [31,32]. In particular, the aggressive arboreal ant *Azteca sericeasur* nests in shade trees, forages on coffee shrubs, and is a keystone predator that controls the CBB [33]. Like many arboreal ants, *A. sericeasur* prefers walking on branches and vegetation to avoid traveling on the ground [27]. Given the role of *A. sericeasur* as a biological control agent, understanding how connectivity at the local scale impacts these ants has potential implications for coffee agroecosystem management. In Chiapas, Mexico, Jiménez-Soto et al. (2019) [21] found that artificially increasing connectivity between *A. sericeasur* nests and coffee plants by tying jute string between ant nest trees and coffee plants increased the capacity for *A. sericeasur* to remove the CBB by throwing them off the coffee plants [21]. These results suggest that naturally occurring vegetation connectivity might have a similar effect as that of artificial string connectivity on *A. sericeasur* activity and their associated pest removal services. Our study tests and expands on this hypothesis by examining the impact of both artificial connectivity (strings) and naturally occurring vegetation connectivity (vines, leaves, and branches) on *A. sericeasur* activity, its ability to recruit to resources, and its removal of the CBB with a manipulative experiment. Specifically, we tested the following hypotheses: We predicted that (a) the coffee plants with vegetation or artificial connections to the ant nest tree have higher *A. sericeasur* activity than that of the isolated control plants; (b) *A. sericeasur* ants recruit to resources more efficiently on coffee plants with vegetation or artificial connections to the nest tree; (c) coffee plants with vegetation or artificial connections to the nest tree have greater CBB removal rates by *A. sericeasur* ants; and (d) *A. sericeasur* activity, resource recruitment rates, and CBB removal rates decrease with increased distance from *A. sericeasur* nests.

## 2. Materials and Methods

### 2.1. Study Site

This study was conducted in the Soconusco region of Chiapas, Mexico at *Finca Irlanda*, a shaded, 300-hectare commercial polyculture coffee plantation. The plantation is located in the Sierra Madre de Chiapas Mountains at an elevation of 1100 m.a.s.l. The average canopy cover throughout the farm is 75 percent and the majority of the plantation shade trees are of the genus *Inga* [21]. The climate is semi-tropical, with the rainy season occurring between May and October. Vegetation management at *Finca Irlanda* frequently includes “*chaporreo*”, in which farmworkers periodically use machetes to clear the weeds and epiphytes that grow between coffee plants. This management practice facilitates farmworker movement between coffee plants and reduces competition between weeds and coffee plants, but in the process inadvertently eliminates vegetation connections between the coffee plants and *A. sericeasur* nest trees.

### 2.2. Field Experiment

We collected data between June and August in the summer of 2022. Within the 300 hectares of *Finca Irlanda*, we selected 17 trees with active *A. sericeasur* nests as study sites. Each site was located at least 10 m away from any other active *A. sericeasur* nests to prevent overlapping ant activity, following the methodology used by Jimenez-Soto et al. (2019) [21].

We chose six coffee plants within a 5 m radius of each *A. sericeasur* nesting tree (each site) for a total of 102 coffee study plants. At each nesting tree site, we selected two coffee plants for the natural vegetation connectivity treatment, two for the artificial connectivity (strings) treatment, and two as isolated control plants (*n* = 2 per treatment per site for each of the 17 sites). For the vegetation connectivity treatment, we selected two coffee plants with existing vegetation connections. The vegetation connections were either (a) coffee branches directly touching the *A. sericeasur* nest tree or (b) coffee branches touching a secondary plant, such as a vine or epiphyte that was touching the nest tree. We selected two coffee plants for the artificial connectivity treatment, in which we tied jute strings (0.95 cm thickness) between the point of the nest tree trunk with the most active ant foraging trail and the central trunk of each coffee plant. We ensured that there were no existing vegetation connections on these plants and that the string was the only point of connection between each coffee plant and the nest tree. For the control treatment, we selected two isolated coffee plants with no connections between the coffee plants and the nest tree. We measured the distance between the central trunk of each study coffee plant and the ant nest tree.

### 2.3. Sampling

#### 2.3.1. Ant Activity

At each site, we quantified the ant activity on the coffee plants by counting the number of *A. sericeasur* that passed a central point on the central trunk of each coffee plant during 1 min (one observation per coffee plant per sampling period) (as in [21]). The observations took place between 7:30 AM and 2 PM before the afternoon rainy period. The observations were stopped if it began to rain, as rain significantly reduces ant activity. After setting up the strings, we returned to each site between 7 and 13 days after the initial setup and re-measured ant activity on the coffee plants.

#### 2.3.2. Pest Removal

To assess the impact of artificial and vegetation connectivity on prey removal by *A. sericeasur*, we placed five dead adult female CBB on white index cards on the central trunk of each coffee plant (as in [21]). We monitored *A. sericeasur* interactions on the cards for one hour, ensuring that only *A. sericeasur* were responsible for removing CBB, and counted the number of CBB removed. Because it has already been well-documented that *A. sericeasur* remove live CBB from coffee plants [33,34], we used dead prey to avoid the possibility of live CBB escaping during a longer observation period. The CBB were collected from infested coffee berries in the field, then frozen for up to 5 days before use.

#### 2.3.3. Resource Recruitment

Recruitment is understood to be an integral component of trail-following in which ant workers follow chemical foraging trails to a food source, then re-apply chemical trails until that food source is exhausted [35]. Tuna baiting is an effective and widely used method of assessing ant recruitment in coffee agroecosystems [36,37]. To assess the impact of connectivity on ant resource recruitment efficiency, we placed 1 g of canned tuna on the central trunk of each coffee plant 1 m above ground and recorded the number of *A. sericeasur* that recruited to each tuna bait after 20 min.

#### 2.3.4. Establishing Foraging Pathways over Time

To assess the impact of time on ant pathfinding learning ability in the context of artificial connectivity, we repeated three rounds of the aforementioned sampling (ant activity, CBB removal rate, and recruitment to resources) 1 week (±5 days), 3 weeks (±4 days), and 5 weeks (±7 days) after the initial setup, with 9–14 days between each sampling round at each site.

### 2.4. Data Analysis

To test for statistical differences in (1) ant activity, (2) resource recruitment efficiency, and (3) CBB removal between the control, string, and vegetation treatment coffee plants over the 5-week experiment, we fit our data with generalized linear mixed models (GLMMs) using the lme4 package in R (version 4.2.0) [38]. For each response variable (ant activity, resource recruitment, and CBB removal), we included the time (the number of weeks post-treatment, which we scaled and centered as a continuous variable for model convergence), the treatment method (control, string, and vegetation), the distance between the coffee plant and the ant nest tree (which we scaled and centered for model convergence), the interaction between the treatment and the time, and the interaction between the time and the distance as fixed effects. As random effects, we modeled the coffee plant identity nested within the site (nest tree identity) to control for site variation and spatial non-independence. To assess count data (our response variable), we originally fit each model to a Poisson distribution. However, to correct for observed over-dispersion, we instead modified each model to a negative binomial distribution.

## 3. Results

We observed *A. sericeasur* using the artificial string connections at 12 of the 17 sites (71%) and on 20 of the 33 strings placed (one string was cut down mid-experiment and not included in the results). *A. sericeasur* were the only ants observed using the strings. Out of 33 vegetation treatment plants (one connection was cut down mid-experiment), 20 plants included primary connections (the coffee plant directly touching the ant nest tree), and 13 plants were connected by secondary connections (coffee branches touching a secondary plant, such as a vine or epiphyte that was touching the nest tree). We observed *A. sericeasur* utilizing vegetation connections on every vegetation treatment plant.

### 3.1. Ant Activity

**Figure 1:**
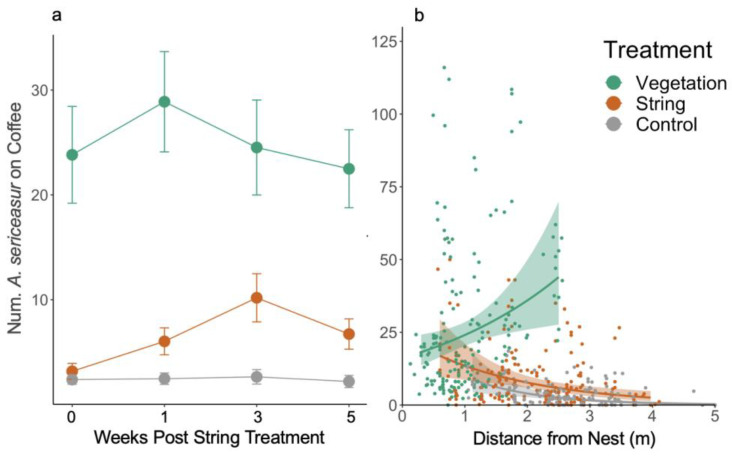
(**a**) *Azteca sericeasur* activity (mean number of ants ± standard error) on control, string, and vegetation coffee plants between the weeks post-string placement. (**b**) *Azteca sericeasur* activity on coffee plants that were not connected to shade trees with ant nests (control: grey), that were connected to shade trees with ant nests with string (orange), and that were connected to trees with ant nests with natural vegetation bridges (green) as a function of distance from the ant nest tree. Each point represents data from one coffee plant and color bands represent 95% confidence bands.

Ant activity was higher on the vegetation treatment coffee plants than on both the control treatment and the string treatment (Figure 1a, Table 1). There was no difference in ant activity between the string and control treatments (Figure 1a, Table 1). There was a significant effect of time on ant activity for the string treatment, indicating an increase in ant activity on the string plants after connecting the strings (Table 1). There was no effect of time on ant activity for either the control or vegetation treatments, indicating that there was no significant change in ant activity for those treatments over the 5-week experiment (Table 1).

The distance between the coffee treatment plants and ant nest trees ranged from 0.3 m to 5 m. The distance from the ant nest tree had a significant negative effect on ant activity for both the control and string treatments (Figure 1b, Table 1). Notably, the distance from the nesting tree had no significant impact on ant activity for the vegetation treatment.

### 3.2. Resource Recruitment

**Figure 2:**
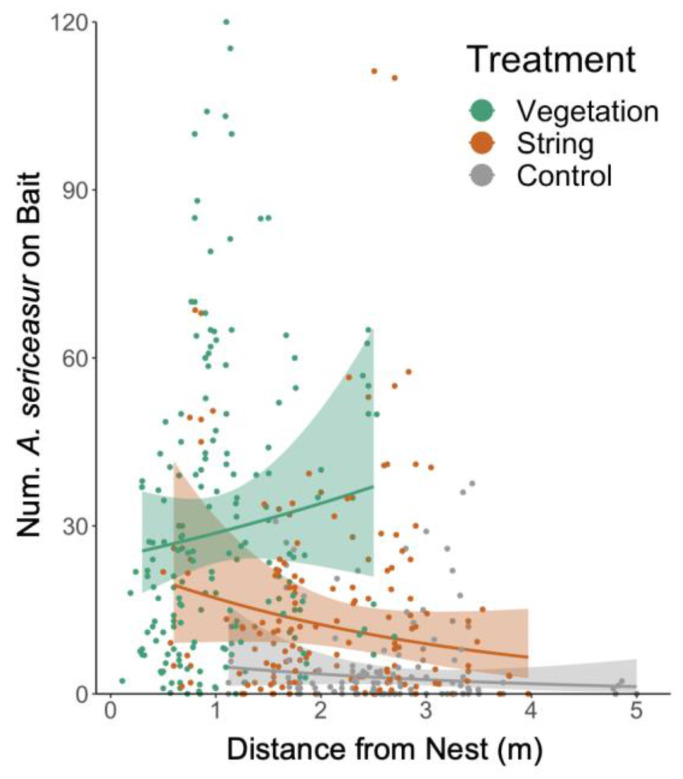
Number of *Azteca sericeasur* that recruited to a tuna bait after 20 min on control, string, and vegetation coffee plants as a function of distance from the ant nest tree. Each point represents data from one coffee plant, and color bands represent 95% confidence bands.

Treatment, time, and distance all impacted the ant recruitment to tuna baits. More ants recruited to the tuna baits on the vegetation treatment plants than on the strings, and more ants recruited to the bait on string plants as compared to the control plants (Figure 2, Table 2). The overall number of ants that recruited to the baits decreased with time post-string placement on both the control and vegetation plants, but there was no significant change in the number of ants recruiting to the baits on the string treatment (Table 2). The number of ants recruiting to the baits on the control and string plants declined with distance from the nest tree, but was consistent over all distances for vegetation treatment plants (Table 2).

### 3.3. Pest Removal

**Figure 3:**
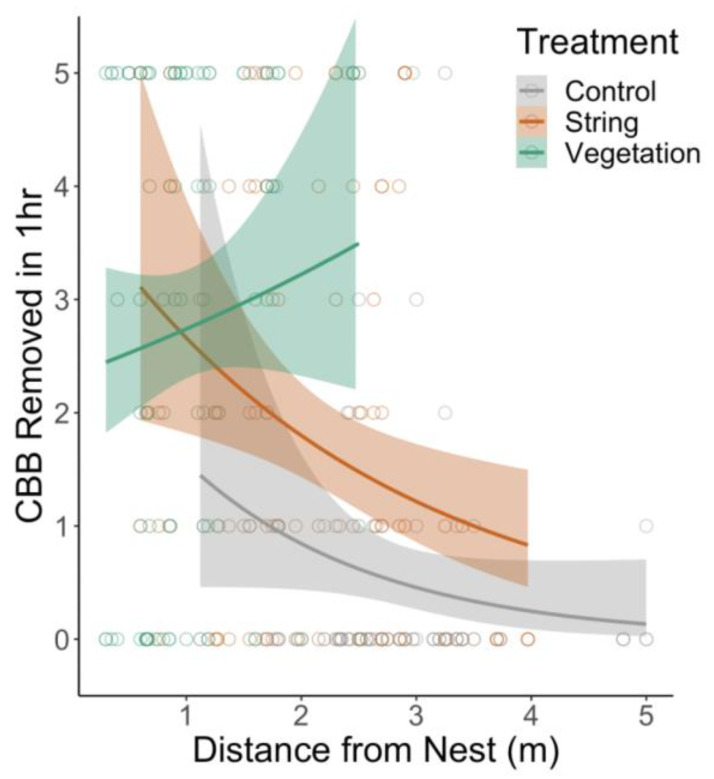
Number of coffee berry borer (CBB) individuals removed in one hour by *Azteca sericeasur* on control, string, and vegetation coffee plants with increasing distance from the shade tree with the *A. sericeasur* nest. Each point represents data from one coffee plant, and color bands represent 95% confidence bands.

Treatment, time, and distance all impacted the number of CBB removed by ants. Ants removed more CBB from the natural vegetation treatment plants than from the string plants and removed more CBB on the string plants compared to the control plants (Figure 3, Table 3). The overall number of CBB removed from the vegetation plants decreased with time post-string placement (Table 3). The number of CBB removed on the control and string plants declined with distance from the nest tree, but was consistent over all distances for vegetation treatment plants (Table 3).

## 4. Discussion

This study asked how connectivity, occurring naturally as vegetation or artificially as string connections, influences *A. sericeasur* activity, foraging efficiency, and pest removal services in coffee systems. Our research demonstrates that naturally occurring vegetation connectivity and, to a lesser extent, artificial connectivity between *A. sericeasur* nest trees and coffee plants increased both *A. sericeasur* mobility and CBB removal on coffee plants. Between the control, string, and vegetation connectivity treatments, all response variables (ant activity, ant recruitment to the baits, and removal of the CBB) were highest on coffee plants with naturally occurring vegetation bridges between the coffee plants and the ant nest tree. Although string connections did increase ant resource recruitment efficiency and pest removal rates compared to the control treatments, *A. sericeasur* exhibited a clear preference for natural vegetation over string connections. Interestingly, while distance from the *A. sericeasur* nest tree did negatively impact ant activity, recruitment to baits, and ant-mediated CBB removal on the control and string treatments, distance did not affect these response variables on the vegetation treatment plants.

Vegetation connectivity influences the distribution, diversity, and interspecific competition of arboreal ant species by affecting the availability of nesting habitats, foraging ranges, and resource availability [26]. Higher degrees of vegetation connectivity provide a range of food resources to arboreal ants, including access to honeydew-producing insects, extrafloral nectaries, and other insects. Arboreal ants can take advantage of these resources more efficiently when connected vegetation provides a network of foraging opportunities, which increases access to patchy resources while enhancing predator avoidance capability [23]. In contrast, disconnected vegetation may limit ant distribution to isolated tree patches [26].

### 4.1. Ant Activity

Observed increases in ant activity on plants with vegetation connectivity suggest that structural connectivity facilitates ant mobility and movement efficiency on foraging paths. Between the control, string, and vegetation treatments, ant activity was highest on vegetatively connected plants (Figure 1a, Table 1). After string placement, ant activity did increase on the string plants, indicating that *A. sericeasur* learned to use strings as foraging paths over time; however, the overall ant activity levels on the string treatment plants were not significantly different from the control treatment. The significant positive interaction between the string treatment and time explains the observed increase in ant activity on the string treatment plants. On the control and vegetation treatment plants, there was no change in ant activity throughout the 5-week duration of the experiment.

Consistent with Jiménez-Soto et al. (2019) [21], ant activity decreased on coffee plants with increasing distance from *A. sericeasur* nest trees on the control and string plants. Notably, our additional treatment of naturally occurring vegetation connectivity overrode the effect of distance from ant nest trees, with no impact of distance on the amount of ant activity on the vegetation treatment plants. This important finding suggests that vegetation pathways can facilitate *A. sericeasur* foraging activity on coffee plants that are farther away from their nest tree. Allowing a longer network of vegetation connectivity could even increase *A. sericeasur* foraging ranges. Additionally, increasing their foraging range may help the ants to avoid parasitic phorid flies in the genus *Pseudacteon*, which parasitize *A. sericeasur* and decrease in density with increasing distance from the *A. sericeasur* nests (observed in our study and consistent with Philpott et al. (2009) [39]).

*A. sericeasur* activity may be highest on coffee plants with naturally occurring vegetation connections because vegetation connections are generally larger and more structurally complex. Additionally, on the existing vegetation pathways, the ants had more time to establish foraging trails and chemical cues as compared to the string connections. Furthermore, in addition to providing linear foraging trails, vegetation bridges may also contain useful resources including extrafloral nectaries and plant fluids [40] that the strings do not provide. Vegetation pathways can also offer protection from phorid flies beneath the leaves, whereas strings are open and unprotected foraging paths.

**Figure 4:**
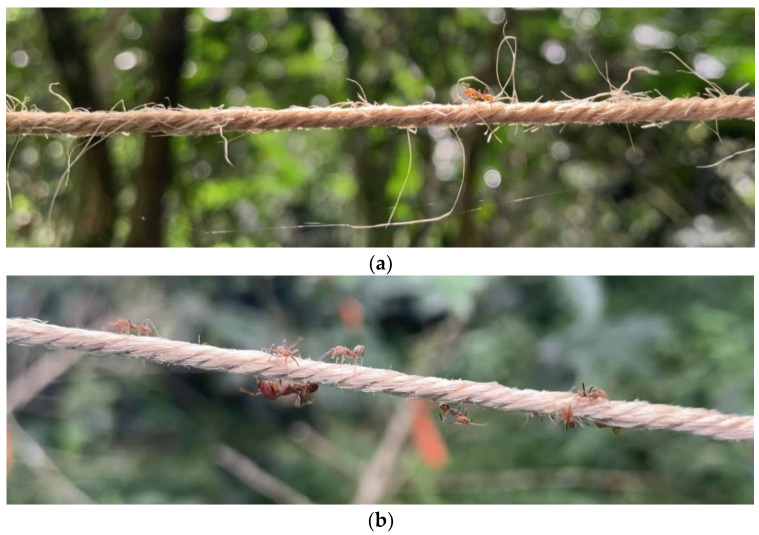
(**a**) *A. sericeasur* on a jute string navigating stray threads. (**b**) *A. sericeasur* on a “cleaned” jute string after ant workers cut off stray threads.

Studies have also suggested that ants have preferences for foraging on certain surfaces, and that surface characteristics impact foraging speed and chemical communication on the ants’ trails [25,41]. The *A. sericeasur* preference for vegetation surfaces may therefore result from texture-based foraging efficiency differences between vegetation and string. Yanoviak et al. (2017) [41] studied ant recruitment to baits on bare vs. moss-covered tree trunk surfaces and observed the *Azteca* spp. actively avoiding baits on moss-covered trunks, indicating a clear surface preference for smoother pathways. In our study, we observed *A. sericeasur* walking around stray threads on the jute strings (Figure 4), decreasing their foraging efficiency compared to smoother thread-free vine surfaces. In some instances, we observed *A. sericeasur* “cleaning” the string pathways by biting off jute string threads from the connections to minimize obstacles and enhance their efficiency on these pathways.

Another explanation for higher *A. sericeasur* activity on the vegetation treatment coffee plants is that *A. sericeasur* may already be tending established green scale (*Coccus viridis*) colonies on vegetatively connected plants, drawing their activity to these plants over the string treatment plants. *C. viridis,* a sessile coffee scale insect, has been linked to increased *A. sericeasur* activity [42]. In a mutualistic relationship, *A. sericeasur* protect *C. viridis* from predation in exchange for the honeydew that these scales produce [43,44,45]. Increased connectivity, by increasing ant mobility, may also increase the scale tending activity by *A. sericeasur*. Notably, interactions between *A. sericeasur* and CBB on coffee plants occur more frequently with higher densities of *C. viridis* [34], indicating a relationship between scale tending activity and CBB control services.

### 4.2. Resource Recruitment

*A. sericeasur* recruited most efficiently to tuna baits on the vegetation treatment plants, and significantly more ants recruited to tuna baits on the string treatment plants than on the control plants (Figure 2, Table 2). The significant difference between control and string treatments found in our resource recruitment results, but not seen in our ant activity results, may have occurred because the ants did not have an incentive to travel to the string treatment plants in the absence of the tuna baits. Our results indicate vegetation connectivity, and, to a lesser extent, artificial connectivity increases the *A. sericeasur* efficiency at discovering and recruiting to resources. In other systems, the ants discover resources more quickly by walking on fallen branches than by traveling through leaf litter [46]; in this case, the fallen branches are a form of vegetation connectivity that advantageously increase colony resource acquisition by reducing the searching effort.

Consistent with the ant activity results, the number of ants recruiting to baits on control and string plants declined with distance from the ant nest tree but remained consistent over all distances for vegetation treatment plants. These results confirm the *A. sericeasur* preference for vegetation foraging paths over artificial ones, as explained in Section 4.1.

Between treatments, the control treatment plants had the lowest ant recruitment to baits. Other ants in tropical systems similarly prefer vegetation pathways over ground and leaf litter, optimizing networks of vines, leaves, and branches in their foraging trails [46]. Clay et al. (2010) [25] suggest that ants may even favor vines over bark or moss because the linear nature of vines reduces the necessity for intensive chemical trail maintenance. Strings might similarly provide this linear path advantage, which reduces chemical trail maintenance and opportunities for path confusion compared to ground trails. Because ants account for energy efficiency when deciding between foraging paths [47], the control plant baits were likely the least attractive because they required the highest energy expenditure due to traveling over ground and leaf litter. Because none of the tuna baits were depleted within the 20 min observation period, recruiting to control baits while more energy-efficient paths were present is an inefficient use of ant workforce.

Over time, the overall number of ants that recruited to the baits decreased with time post-string placement on both the control and vegetation plants, but there was no significant change in the number of ants that recruited to the baits on the string treatment. Decreases in ant recruitment rates on the control and vegetation treatment plants may have resulted from the presence of phorid flies, which greatly reduce *A. sericeasur* activity [48]. Phorid fly attacks may have curtailed ant recruitment along the pre-existing foraging routes, as phorids are likely more abundant in leaf litter along popular foraging routes. Because the strings were a novel foraging route, it is likely that fewer phorids frequent those routes and interfere less with ant recruitment to the baits.

### 4.3. Pest Removal

Between treatments, *A. sericeasur* removed the most CBB from vegetation treatment plants and removed more CBB on the string plants as compared to the control plants (Figure 3, Table 3). The overall number of CBB removed on the vegetation plants decreased with time post-string placement. Decreases in CBB removal may similarly have resulted from phorid fly attacks inhibiting pest removal activity, as occurred in Philpott et al. (2004) [48] and Pardee and Philpott (2011) [49]. Consistent with the results of our ant activity and resource recruitment experiments, the number of CBB removed on the control and string plants declined with distance from the nest tree, but remained consistent with distance from the nest on the vegetation treatment plants. Interestingly, Jiménez-Soto et al. (2019) [21] did not find any effect of distance from the nest tree on CBB removal for the control or string plants. Our contrasting results may be the result of the *A. sericeasur* preference for the vegetatively connected plants in our study; in the absence of vegetation pathways, ants may forage more on artificial connections. Our results reinforce how habitat complexity in the form of vegetation connectivity impacts interspecific interactions, specifically ant-mediated CBB removal at the local scale.

### 4.4. Management Implications

Our results confirm the importance of naturally occurring vegetation connectivity and habitat complexity in facilitating arboreal ant mobility and ant-mediated CBB removal. Our findings have important implications for the practical application of ant-provided pest removal in coffee systems, indicating that *A. sericeasur* may most effectively control CBB on coffee plants with natural vegetation connectivity connected to their nest trees. In the absence of vegetation connectivity, implementing artificial connections between ant nests and coffee plants can increase CBB removal by *A. sericeasur*; however, with increasing distance from the ant nest tree, the strength of this pest removal service decreases on artificially connected plants. The observed preference of *A. sericeasur* for vegetation pathways underscores the importance of maintaining or promoting vegetation connectivity via habitat complexity and structural diversity within coffee agroecosystems. In managing agroecosystems in support of ant-mediated ecosystem services, artificial connectivity does not provide an equal substitute for the naturally occurring vegetation connectivity provided through forest conservation and structural complexity.

Consistent with studies affirming the influence of vegetation connectivity on predatory arthropod movement and predation range [50,51], our results illustrate how vegetation connectivity facilitates *A. sericeasur* foraging mobility and pest removal. In coffee systems, higher degrees of vegetation connectivity are associated with shade trees, as well as more heterogeneous habitat complexity and variability in plant structure. In other studies, ants generally increase predation services in shaded systems as compared to monocultures [11] and, in coffee plants, more effectively remove CBB in shaded coffee systems as compared to sun monoculture systems [52]. Interestingly, most studies find the opposite effect of structural complexity on parasitoid behavior, with higher degrees of plant structural complexity leading to decreased parasitoid foraging efficiency [53,54]. This negative relationship between parasitism and habitat complexity transfers to coffee systems, where the parasitic phorid flies exert a greater inhibiting effect on *Azteca* ants in simple, low-shade farms than in complex, high-shade farms [49]. Together with the aforementioned study, our combined results illustrate how habitat complexity at the landscape scale and vegetation connectivity at the plot scale dually facilitate *A. sericeasur-*mediated pest removal: by facilitating ant mobility and by reducing the efficiency of the parasitoid that interferes with their pest removal ability.

In order for *A. sericeasur* to provide ant-mediated pest removal services, coffee agroforests must include enough shade trees to provide sufficient habitats for ant nests. Planting coffee plants close enough to shade trees to allow for direct connectivity and leaving some vegetation connections between coffee plants and shade trees rather than chopping them or relying on herbicides can facilitate ant-provided ecosystem services by providing foraging paths through naturally occurring structural connectivity. By enhancing the *A. sericeasur* effectiveness in controlling CBB populations, vegetation connectivity can potentially reduce chemical pesticide use.

Our results offer management insight into one piece of a complex ecological puzzle. Because *A. sericeasur* tend *C. viridis* [42,55], they could indirectly reduce coffee plant growth by contributing to high-scale densities and an associated damaging sooty mold. However, high densities of *C. viridis* also beneficially attract *Lecanicillium lecanii* [56], which attacks coffee leaf rust (*Hemileia vastatrix*) [57,58,59], a devastating coffee fungal disease. Moreover, the CBB is regarded as a far more damaging coffee pest than *C. viridis* [60].

Furthermore, facilitating the mobility of *A. sericeasur* as a single ant species is not necessarily the most effective pest management approach, as higher ant diversity can improve pest control through the cooperation of complementary predatory species [61,62]. Enhanced *A. sericeasur* activity on coffee plants could alter the behavior of other ant species, which could have positive or negative effects on overall pest control services due to spatial complementarity or potential negative interactions between predators [34]. However, studies find that increasing connectivity generally increases species richness [22,23,26,27], and so, vegetation connections that increase *A. sericeasur* mobility likely facilitate the mobility of other predatory ants in coffee systems, even by providing alternative paths to avoid aggressive altercations with *A. sericeasur*. Although *A. sericeasur* occupies only 3–5% of the shade trees at our research site [63], other ants known to contribute to CBB regulation would likely also use vegetation pathways, facilitating additional pest control. Future research should examine how vegetation connectivity impacts the abundance and diversity of other ant species on coffee plants and the associated spatial complementarity between specific predators of the CBB. Future studies could also investigate how phorid attacks on *Azteca* vary on different foraging pathways to better understand the mechanisms behind their preference for vegetation pathways.

## 5. Conclusions

Connectivity affects arboreal ant distribution, behavior, and interactions with other organisms in agroecosystems, profoundly impacting ant community diversity and ant-provided ecosystem services [25,64]. Our results demonstrate how vegetation connectivity increases *A. sericeasur* activity, recruitment to resources, and CBB removal, and that naturally occurring vegetation connectivity, in the form of branches and natural substrates, accounts for this enhancement. As climate change increases coffee’s susceptibility to CBB damage [65,66], agroecological and economically feasible forms of pest control are increasingly necessary for coffee-producing communities. Farm management conducive to forest conservation, habitat and structural complexity, and the associated higher degrees of vegetation connectivity will facilitate ant-provided pest control services in coffee agroecosystems.

## Figures and Tables

**Table 1:** Model results for our generalized linear mixed model of ant activity on coffee plants with parameter estimates, standard error, Wald Z scores, and *p*-values.

Fixed Effects	Estimate	Std Error	*Z* Value	Pr(>|*z*|)
Reference: Treatment (Control)				
(Intercept)	**1.030**	**0.300**	**3.435**	**<0.001**
TreatmentVegetation	**2.138**	**0.440**	**4.862**	**<0.001**
TreatmentString	0.250	0.312	0.800	0.424
Time	−0.062	0.082	−0.758	0.449
Distance	**−1.291**	**0.307**	**−4.204**	**<0.001**
TreatmentVegetation:Time	0.013	0.098	0.136	0.891
TreatmentString:Time	**0.372**	**0.109**	**3.423**	**<0.001**
TreatmentVegetation:Distance	**1.648**	**0.435**	**3.787**	**<0.001**
TreatmentString:Distance	0.265	0.375	0.707	0.480
Reference: Treatment (String)				
(Intercept)	**1.279**	**0.230**	**5.572**	**<0.001**
TreatmentControl	−0.250	0.312	−0.800	0.424
TreatmentVegetation	**1.888**	**0.388**	**4.867**	**<0.001**
Time	**0.310**	**0.072**	**4.316**	**<0.001**
Distance	**−1.026**	**0.250**	**−4.101**	**<0.001**
TreatmentControl:Time	**−0.372**	**0.109**	**−3.423**	**<0.001**
TreatmentVegetation:Time	**−0.359**	**0.090**	**−3.974**	**<0.001**
TreatmentControl:Distance	−0.265	0.375	−0.707	0.480
TreatmentVegetation:Distance	**1.384**	**0.402**	**3.438**	**<0.001**
Reference: Treatment (Vegetation)				
(Intercept)	**3.167**	**0.373**	**8.481**	**<0.001**
TreatmentString	**−1.888**	**0.388**	**−4.867**	**<0.001**
TreatmentControl	**−2.138**	**0.440**	**−4.862**	**<0.001**
Time	−0.048	0.055	−0.887	0.375
Distance	0.357	0.322	1.108	0.268
TreatmentString:Time	**0.359**	**0.090**	**3.974**	**<0.001**
TreatmentControl:Time	−0.013	0.098	−0.136	0.891
TreatmentString:Distance	**−1.384**	**0.402**	**−3.438**	**<0.001**
TreatmentControl:Distance	**−1.648**	**0.435**	**−3.787**	**<0.001**

All significant factors and interactions (shown in bold) were significant at the level of *p* < 0.001.

**Table 2:** Model results for our generalized linear mixed model of the number of ants that recruited to tuna baits on coffee plants with parameter estimates, standard error, Wald Z scores, and *p-*values.

Fixed Effects	Estimate	Std Error	*Z* Value	Pr(>|*z*|)
Reference: Treatment (Control)				
(Intercept)	0.509	0.471	1.082	0.279
TreatmentVegetation	**2.528**	**0.654**	**3.867**	**<0.001**
TreatmentString	**0.961**	**0.467**	**2.059**	**0.039**
Time	**−0.493**	**0.191**	**−2.576**	**0.010**
Distance	**−1.198**	**0.426**	**−2.815**	**0.005**
TreatmentVegetation:Time	0.247	0.228	1.086	0.278
TreatmentString:Time	**0.612**	**0.240**	**2.549**	**0.011**
TreatmentVegetation:Distance	1.187	0.606	1.959	0.050
TreatmentString:Distance	−0.106	0.519	−0.205	0.838
Reference: Treatment (String)				
(Intercept)	**1.470**	**0.357**	**4.121**	**<0.001 **
TreatmentControl	**−0.961**	**0.467**	**−2.060**	**0.039**
TreatmentVegetation	**1.567**	**0.558**	**2.809**	**0.005**
Time	0.119	0.145	0.819	0.413
Distance	**−1.304**	**0.367**	**−3.549**	**<0.001 **
TreatmentControl:Time	**−0.612**	**0.240**	**−2.549**	**0.011**
TreatmentVegetation:Time	−0.364	0.191	−1.913	0.056
TreatmentControl:Distance	0.106	0.519	0.205	0.838
TreatmentVegetation:Distance	**1.294**	**0.581**	**2.226**	**0.026**
Reference: Treatment (Vegetation)				
(Intercept)	**3.037**	**0.550**	**5.520**	**<0.001 **
TreatmentString	**−1.567**	**0.558**	**−2.809**	**0.005**
TreatmentControl	**−2.528**	**0.654**	**−3.867**	**<0.001 **
Time	**−0.246**	**0.124**	**−1.983**	**0.047**
Distance	−0.010	0.461	−0.023	0.982
TreatmentString:Time	0.364	0.191	1.913	0.056
TreatmentControl:Time	−0.247	0.228	−1.086	0.278
TreatmentString:Distance	**−1.294**	**0.581**	**−2.226**	**0.026**
TreatmentControl:Distance	−1.187	0.606	−1.959	0.050

Significant (*p* < 0.05) model terms are shown in bold.

**Table 3:** Model results for our generalized linear mixed model of the number of coffee berry borer (CBB) individuals removed in one hour by *Azteca sericeasur* on control, string, and vegetation coffee plants with parameter estimates, standard error, Wald Z scores, and *p-*values.

Fixed Effects:	Estimate	Std. Error	*Z* Value	Pr(>|*z*|)
Reference: Treatment (Control)				
(Intercept)	−0.244	0.254	−0.960	0.337
TreatmentVegetation	**1.250**	**0.331**	**3.780**	**<0.001 **
TreatmentString	**0.621**	**0.258**	**2.408**	**0.016**
Time	−0.238	0.182	−1.307	0.191
Distance	**−0.779**	**0.247**	**−3.147**	**0.002**
TreatmentVegetation:Time	−0.081	0.204	−0.397	0.691
TreatmentString:Time	0.209	0.212	0.985	0.324
TreatmentVegetation:Distance	**0.841**	**0.326**	**2.580**	**0.010**
TreatmentString:Distance	0.259	0.293	0.885	0.376
Reference: Treatment (String)				
(Intercept)	**0.377**	**0.175**	**2.153**	**0.031**
TreatmentControl	**−0.621**	**0.258**	**−2.410**	**0.016**
TreatmentVegetation	**0.629**	**0.271**	**2.320**	**0.020**
Time	−0.030	0.107	−0.276	0.782
Distance	**−0.520**	**0.182**	**−2.854**	**0.004**
TreatmentControl:Time	−0.208	0.211	−0.983	0.325
TreatmentVegetation:Time	**−0.289**	**0.140**	**−2.063**	**0.039**
TreatmentControl:Distance	−0.259	0.293	−0.885	0.376
TreatmentVegetation:Distance	**0.582**	**0.282**	**2.067**	**0.039**
Reference: Treatment (Vegetation)				
(Intercept)	**1.007**	**0.263**	**3.822**	**<0.001 **
TreatmentString	**−0.630**	**0.271**	**−2.320**	**0.020**
TreatmentControl	**−1.250**	**0.331**	**−3.779**	**<0.001 **
Time	**−0.319**	**0.091**	**−3.526**	**<0.001 **
Distance	0.063	0.223	0.281	0.778
TreatmentString:Time	**0.290**	**0.141**	**2.058**	**0.040**
TreatmentControl:Time	0.080	0.204	0.395	0.693
TreatmentString:Distance	**−0.582**	**0.282**	**−2.067**	**0.039**
TreatmentControl:Distance	**−0.841**	**0.326**	**−2.580**	**0.010**

Significant (*p* < 0.05) model terms are shown in bold.

## Data Availability

The summary data used for this article is available on Dryad. DOI: 10.5061/dryad.8931zcrxd.
